# The impact of hepatic steatosis on portal hypertension

**DOI:** 10.1371/journal.pone.0224506

**Published:** 2019-11-06

**Authors:** Georg Semmler, Bernhard Scheiner, Philipp Schwabl, Theresa Bucsics, Rafael Paternostro, David Chromy, Albert Friedrich Stättermayer, Michael Trauner, Mattias Mandorfer, Arnulf Ferlitsch, Thomas Reiberger

**Affiliations:** 1 Division of Gastroenterology and Hepatology, Department of Internal Medicine III, Medical University of Vienna, Vienna, Austria; 2 Vienna Hepatic Hemodynamic Laboratory, Medical University of Vienna, Vienna, Austria; 3 Department of Internal Medicine I, Hospital of St. John of God, Vienna, Austria; Medizinische Fakultat der RWTH Aachen, GERMANY

## Abstract

**Background and aims:**

Studies in animal models have suggested that hepatic steatosis impacts on portal pressure, potentially by inducing liver sinusoidal endothelial dysfunction and thereby increasing intrahepatic resistance. Thus, we aimed to evaluate the impact of hepatic steatosis on hepatic venous pressure gradient (HVPG) in patients with chronic liver disease.

**Method:**

261 patients undergoing simultaneous HVPG measurements and controlled attenuation parameter (CAP)-based steatosis assessment were included in this retrospective study.

**Results:**

The majority of patients had cirrhosis (n = 205; 78.5%) and n = 191 (73.2%) had clinically significant portal hypertension (CSPH; HVPG≥10mmHg). Hepatic steatosis (S1/2/3; CAP ≥248dB/m) was present in n = 102 (39.1%). Overall, HVPG was comparable between patients with vs. without hepatic steatosis (15.5±7.5 vs. 14.8±7.7mmHg; p = 0.465). Neither in patients with HVPG (<6mmHg; p = 0.371) nor in patients with mild portal hypertension (HVPG 6–9mmHg; p = 0.716) or CSPH (HVPG≥10mmHg; p = 0.311) any correlation between CAP and HVPG was found. Interestingly, in patients with liver fibrosis F2/3, there was a negative correlation between CAP and HVPG (Pearson’s ρ:-0.522; p≤0.001). In multivariate analysis, higher CAP was an independent ‘protective’ factor for the presence of CSPH (odds ratio [OR] per 10dB/m: 0.92, 95% confidence interval [CI]:0.85–1.00; p = 0.045), while liver stiffness was associated with the presence of CSPH (OR per kPa: 1.26, 95%CI: 1.17–1.36; p≤0.001). In 78 patients, in whom liver biopsy was performed, HVPG was neither correlated with percentage of histological steatosis (p = 0.714) nor with histological steatosis grade (p = 0.957).

**Conclusion:**

Hepatic steatosis, as assessed by CAP and liver histology, did not impact on HVPG in our cohort comprising a high proportion of patients with advanced chronic liver disease. However, high CAP values (i.e. pronounced hepatic steatosis) might lead to overestimation of liver fibrosis by ‘artificially’ increasing transient elastography-based liver stiffness measurements.

## Introduction

Non-alcoholic fatty liver disease (NAFLD) is becoming the most prevalent liver disease with 17–46% of adults affected in Western countries.[1] NAFLD is the hepatic manifestation of the metabolic syndrome and may progress to liver fibrosis, cirrhosis, portal hypertension (PHT), and hepatocellular carcinoma often resulting in the need for liver transplantation.[2–5] With increasing severity of liver fibrosis and cirrhosis, portal hypertension (hepato-venous pressure gradient (HVPG) ≥6 mmHg) might develop and progress to clinically significant portal hypertension (CSPH, HVPG ≥10 mmHg).[6] CSPH is the main driver for the development of complications such as variceal bleeding[7], ascites[8] and portosystemic encephalopathy.[6, 9–11] While the role of liver fibrosis as a mechanical resistance factor for development of PHT is well established, the influence of hepatic steatosis needs further investigation. Francque et al. (2010) demonstrated that a rat model of steatohepatitis induced by methionine-choline-deficient diet (MCDD) develops PHT in the absence of fibrosis.[12] These results were confirmed in another animal model of diet-induced obesity and metabolic syndrome: Hepatic steatosis induced liver sinusoidal endothelial dysfunction which in turn increased intrahepatic vascular resistance, and thus, portal perfusion pressure even in the absence of liver fibrosis or hepatic inflammation.[13] Another in-situ perfusion model in rats fed with MCDD showed a distortion of the regular hepatic sinusoidal anatomy by a disorganized pattern with multiple interconnections and vascular extensions. Hepatic overexpression of thromboxane synthase and endothelin-1 were found and the authors suggested sinusoidal endothelial dysfunction as main mechanism responsible for increased intrahepatic vascular resistance.[14] Moreover, a recent animal model confirmed elevated portal pressure and hyperdynamic systemic circulation in a rat-model with severe steatosis.[15] Human evidence for an impact of ‘simple’ steatosis on portal pressure is limited to a small study including non-cirrhotic NAFLD patients reporting an association of steatosis and elevated portal pressure. The degree of steatosis was the only factor being significantly different between patients with HVPG ≤5 mmHg and ≥6mmHg.[16] Metabolic liver disease and hepatic steatosis might also influence HVPG in patients after eradication of hepatitis C virus infection.[17] In summary, these findings suggest that hepatic steatosis may impact on sinusoidal structure and function and thus, on portal pressure. Hepatic steatosis may be best evaluated by liver biopsy, however, this procedure is associated with a series of complications and sampling variabilty.[18–20] Although hepatic steatosis can be accurately quantified by magnetic resonance imaging, the applicability of this method is limited by its resource-intensiveness.[21, 22] In 2010, transient elastography (TE)-based controlled attenuation parameter (CAP) was introduced allowing the assessment of hepatic steatosis at the time of liver stiffness (LSM).[23] Consecutively, the accuracy of CAP for diagnosing hepatic steatosis has been confirmed in several liver biopsy controlled studies.[24–27] Thus, we aimed to clarify the impact of hepatic steatosis assessed by TE-based CAP on HVPG in patients with chronic liver disease.

## Methods

### Patients and definitions

All patients undergoing both HVPG and TE-based CAP measurement between 01.01.2014 and 31.12.2016 at the Medical University of Vienna were included in this retrospective analysis. Exclusion criteria were: (i) unreliable HVPG measurement (presence of intrahepatic veno-venous communications, technical problems, measurement during non-selective β-blocker intake, presence of portal venous thrombosis or Budd-Chiari-Syndrome) or CAP measurement (food intake within 6 hours prior to measurement or technical problems), (ii) previous liver transplantation or transjugular intrahepatic portosystemic shunt (TIPS), (iii) hepatocellular carcinoma (HCC), (iv) acute liver failure or (v) antiviral treatment of viral hepatitis C. If patients underwent more than one HVPG and CAP measurement, only the first adequate measurement was included. Patient characteristics and laboratory parameters were assessed by chart review. Since no clear distinction between alcohol and non-alcoholic fatty liver disease could be made due to the retrospective nature of our study, we combined these two etiologies in one liver disease category.

### HVPG measurements

HVPG measurements were performed at the Vienna Hepatic Hemodynamic Laboratory according to a standardized procedure using a 7F balloon catheter (Pejcl Medizintechnik, Austria) as previously described.[28] In brief, the catheter introducer sheet was placed in the right internal jugular vein under local anesthesia and ultrasound guidance using the Seldinger technique. A balloon catheter was then placed under fluoroscopic control in a large hepatic vein. HVPG was calculated as the difference between wedged hepatic vein pressure and free hepatic vein pressure as a mean of three measurements. Treatment with non-selective β-blockers was paused at least three days prior to the measurement.[29, 30] PHT was defined as HVPG ≥6mmHg, while HVPG values ≥10mmHg denoted CSPH, respectively.

### Liver stiffness and CAP measurements

CAP measurements were done simultaneously to LSM using FibroScan^®^ (Echosense, Paris, France) as previously described.[31] M and XL-probe were used according to the recommendation of the device. Reliability of LSM was determined according to established criteria.[32] Published etiology-specific LSM cut-offs were applied to stage fibrosis,[33–36] CAP cut-offs for differentiating steatosis grades and CAP-corrections were applied according to Karlas et al. (2017)[25]: 10 dB/m were deducted if NAFLD was the underlying liver disease and in patients with diabetes. Moreover, 4.4dB/m were added/subtracted for every body-mass-index (BMI) unit above/below 25 kg/m^2^ within a range of 20–30 kg/m^2^. CAP cut-offs for any steatosis (S1), moderate steatosis (S2), and severe steatosis (S3) were ≥248 dB/m, ≥268 dB/m, and ≥280 dB/m, respectively.

### Liver biopsy

Histological specimens were included in this study, if biopsy was performed within one week after CAP/HVPG measurement using either the transjugular or percutaneous route.[37] Data on fibrosis stage and hepatic steatosis (%) were extracted from medical records. Of note, liver fibrosis was graded according to the Batts-Ludwig or METAVIR score, as applicable.[38, 39] Hepatic steatosis was semi-quantitatively assessed and graded as no hepatic steatosis (S0, fat accumulation in <5% of hepatocytes), grade 1 (S1, fat accumulation in 5–33% of hepatocytes), grade 2 (S2, fat accumulation in 34–66% of hepatocytes), and grade 3 (S3, fat accumulation in ≥67% of hepatocytes).[40]

### Statistics

Statistical analyses were performed using IBM SPSS Statistics 25 (SPSS Inc., Armonk, New York, USA) and GraphPad Prism 6 (GraphPad Software, La Jolla, California, USA). Continuous variables were reported as mean ±standard deviation (SD) or median (interquartile range; IQR), and categorical variables were shown as numbers (n) and proportions (%) of patients. Comparisons of continuous variables were performed using Student *t* test or Mann-Whitney-U test, as applicable. Group comparisons of categorical variables were performed using either Pearson’s chi-squared or Fisher’s exact test. Pearson correlation coefficient was calculated to assess a potential correlation between HVPG and CAP. Otherwise, Spearman’s rank correlation coefficient was used. Univariate and multivariate binary logistic regression analysis was used to determine factors independently associated with the presence of CSPH. A p-value ≤0.05 was considered statistically significant.

### Ethics

This study was approved by the ethics committee of the Medical University of Vienna (EK: 1124/2017) and performed in accordance with the ethical guidelines denoted in the Declaration of Helsinki (version 2008). As this study is a retrospective analysis and according to the Ethic’s vote no written informed consent was required. Patients’ data were pseudo-anonymized before inclusion in this study.

## Results

### Patient characteristics (Table 1)

**Table 1:** Comparison of baseline characteristics of patients with vs. without any hepatic steatosis (as according to CAP values).

	All patients, n = 261	No hepatic steatosis (S0), n = 159	Any hepatic steatosis (≥S1), n = 102	p-value
**Sex, male/female (% male)**	166/95 (63.6)	99/66 (62.3)	67/35 (65.7)	0.575
**Age, years**	52.8±11.8	53.1±12.1	52.3±11.1	0.436
**BMI, kg x m**^**-2**^	25.4±4.9	24.8±4.8	26.2±5.1	**0.028**
**Etiology**				
***- (N)AFLD*, *n (%)***	*88 (33*.*7)*	*51 (32*.*1)*	*37 (36*.*3)*	*0*.*077*
***- Cholestatic*, *n (%)***	*12 (4*.*6)*	*11 (6*.*9)*	*1 (1*.*0)*	
***- Viral hepatitis*, *n (%)***	*124 (47*.*5)*	*70 (44*.*0)*	*54 (52*.*9)*	
***- Other*, *n (%)***	*16 (6*.*1)*	*11 (6*.*9)*	*5 (4*.*9)*	
***- Unknown*, *n (%)***	*30 (11*.*5)*	*20 (12*.*6)*	*10 (9*.*8)*	
**Diabetes, n *(%)***	40 *(15*.*3)*	23 *(14*.*5)*	17 *(16*.*7)*	0.827
**Fasting glucose, mg/dL**	101±20	100±22	104±15	0.507
**Triglycerides, mg/dL**	92.6±45.0	91.9±47.0	93.8±42.0	0.737
**Total cholesterol, mg/dL**	146.3±46.5	147.7±48.1	144.2±44.2	0.558
**HIV co-infection, n *(%)***	42 *(16*.*1)*	21 *(13*.*2)*	21 *(20*.*6)*	0.105
**MELD, points**	10±4	11±4	10±4	0.285
**Liver stiffness, kPa (IQR)**	28.0 (14.2–55.1)	28.7 (13.4–51.0)	26.7 (14.4–63.9)	0.532
***- F0/1*, *n (%)***	13 *(5*.*0)*	11 *(6*.*9)*	2 *(2*.*0)*	0.313
***- F2*, *n (%)***	23 *(8*.*8)*	13 *(8*.*2)*	10 *(9*.*8)*	
***- F3*, *n (%)***	20 *(7*.*7)*	13 *(8*.*2)*	7 *(6*.*9)*	
***- F4*, *n (%)***	205 *(78*.*5)*	122 *(76*.*7)*	83 *(81*.*4)*	
**CAP, dB/m**	237±57	200±34	294±33	**<0.001**
**HVPG, mmHg**	15.2±7.5	15.5±7.5	14.8±7.7	0.465
***- CSPH*, *n (%)***	191 (73.2)	118 (74.2)	73 (71.6)	0.638

*Abbreviations*: (N)AFLD (non-)alcoholic fatty liver disease; CAP controlled attenuation parameter; BMI body mass-index; HIV human immunodeficiency virus; MELD model for end-stage liver disease; HVPG hepatic venous pressure gradient; CSPH clinically significant portal hypertension.

Overall, 475 patients underwent simultaneous CAP/LSM and HVPG measurement within the study period. Eighty-nine patients were excluded due to unreliable results of HVPG or CAP measurements, 53 patients due to antiviral therapy of hepatitis C, 45 patients due to previously diagnosed HCC and 20 due to previous liver transplantation or TIPS. Seven patients were excluded due to other reasons (S1 Fig). Finally, 261 patients were included in this retrospective analysis. The majority of patients were male (n = 166, 63.6%) with a mean age of 52.8±11.8 years and a mean BMI of 25.4±4.9 kg/m^2^. Underlying etiologies of liver disease were viral hepatitis in 124 patients (47.5%; including 14 patients with hepatitis B-virus infection and 110 patients with hepatitis C-virus infection), (non)-alcoholic fatty liver disease ([N]AFLD) in 88 patients (33.7%), and cholestatic liver disease in 12 patients (4.6%). In 26 patients, etiology remained cryptogenic while 13 patients had other causes of chronic liver disease. 176 patients (67.4%) had Child-Pugh-Score A, 72 (27.6%) Child-Pugh-Score B and 10 (3.8%) Child-Pugh-Score C with a mean MELD Score of 10±4 points. Median liver stiffness was 28.0kPa (IQR: 14.2–55.1) with liver fibrosis grade F0/1 detected in 13 patients (5.0%), F2 in 23 (8.8%), F3 in 20 (7.7%) and F4 in 205 patients (78.5%), respectively. Mean CAP was 237±57dB/m and mean HVPG 15.2±7.5 mmHg resulting in 191 patients (73.2%) with CSPH at presentation. According to CAP, 159 patients (60.9%) had no steatosis (S0) while 102 patients (40.1%) had any steatosis (≥S1). Except for higher BMI (24.8±4.8 vs. 26.2±5.1 kg/m^2^, p = 0.028), no other baseline characteristics were associated with (any) hepatic steatosis on CAP.

### Influence of baseline factors on CAP values

In order to evaluate baseline factors associated with CAP levels, correlations between CAP and baseline characteristics were investigated. Notably, CAP levels at baseline did neither correlate with age (ρ = -0.034, p = 0.582) nor with BMI (ρ = 0.107, p = 0.086), liver stiffness (ρ = 0.066, p = 0.323), markers of hepatic inflammation (AST (ρ = -0.023, p = 0.710), ALT (ρ = 0.011, p = 0.862), GGT (ρ = 0.100, p = 0.110)), or total cholesterol (ρ = -0.065, p = 0.307), but weakly with triglycerides (ρ = 0.126, p = 0.047). However, before applying ‘CAP-correction’ as proposed by Karlas et al. (2017)[25], a higher BMI was significantly associated with an increase in CAP value (ρ = 0.348, p≤0.001).

### Correlation between CAP and HVPG (Table 2, Fig 1)

**Fig 1:**
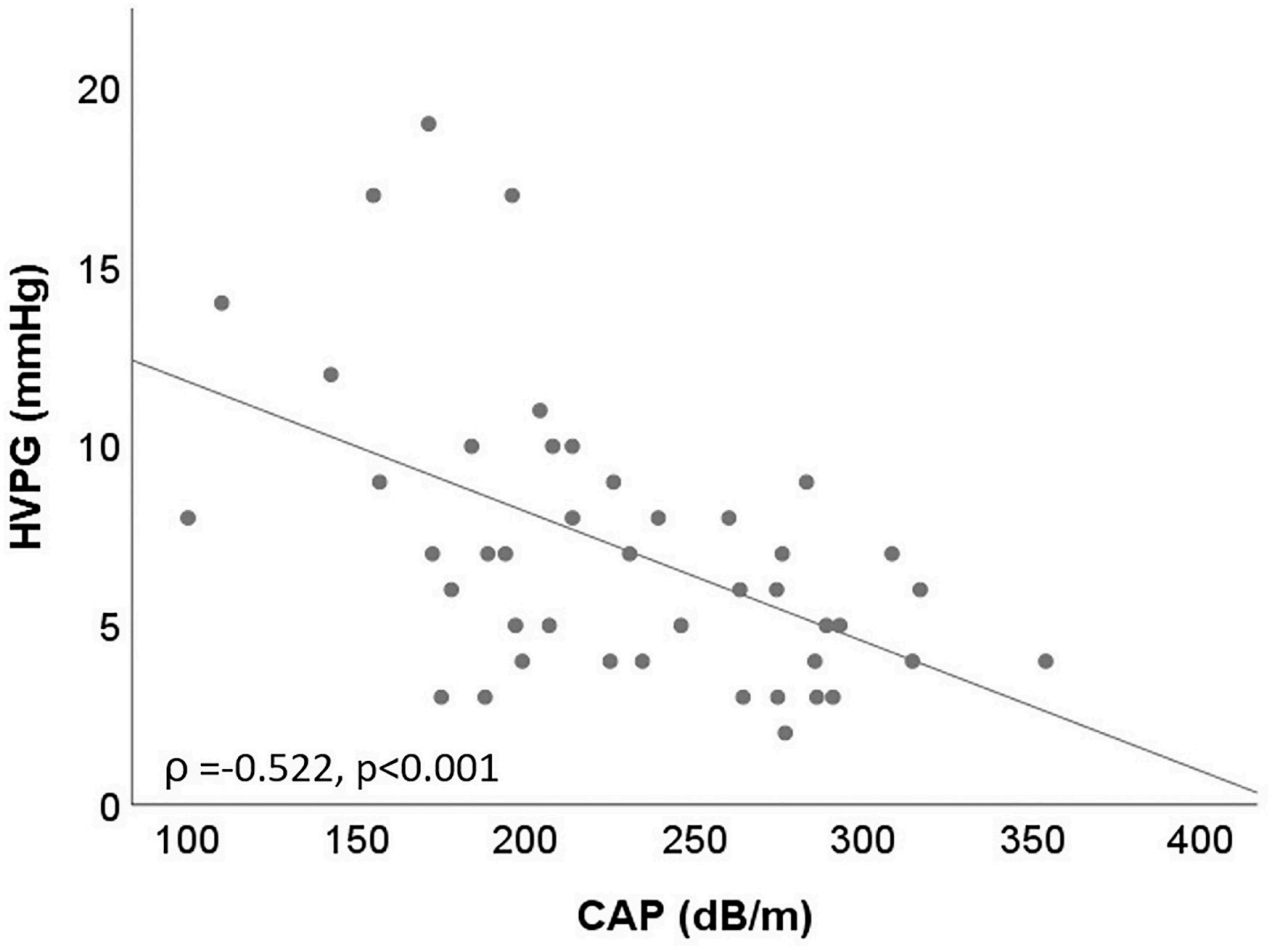
Correlation of CAP and HVPG in patients with F2/F3 fibrosis.

**Table 2:** Correlation between CAP and HVPG in subgroup analyses stratified by the severity of portal hypertension, different etiologies of liver disease and subgroups of different fibrosis stages (as according to transient elastography).

HVPG–CAP	Correlation coeff. (Pearson)	p-value
**1–5 mmHg (n = 36)**	0.154	0.371
**6–9 mmHg (n = 34**	-0.065	0.716
**>10 mmHg (n = 191)**	-0.074	0.311
**(N)AFLD (n = 88)**	0.014	0.898
**Cholestatic liver disease (n = 12)**	0.103	0.750
**Viral liver disease (n = 124)**	**-0.355**	**<0.001**
**Liver disease of unknown origin (n = 30)**	0.189	0.316
**Other known liver diseases (n = 16)**	0.097	0.721
**0/1 (n = 13)**	0.113	0.712
**F2 (n = 23)**	**-0.586**	**0.003**
**F3 (n = 20)**	**-0.543**	**0.013**
**F4 (n = 205)**	**-0.139**	**0.047**
**F2/F3 (n = 43)**	**-0.522**	**<0.001**

*Abbreviations*: HVPG hepatic venous pressure gradient; CAP controlled attenuation parameter; (N)AFLD (non-)alcoholic fatty liver disease;

In the overall cohort, CAP was not significantly correlated with HVPG (Pearson’s ρ = -0.085, p = 0.173). After stratifying patients into subgroups according to different levels of HVPG, there were no associations between CAP-based steatosis and HVPG in patients with normal HVPG (ρ = 0.154, p = 0.371), subclinical portal hypertension (ρ = -0.065, p = 0.716), or CSPH (ρ = -0.074, p = 0.311). When investigating the relationship between CAP and HVPG in different liver disease etiologies, no associations were observed in (N)AFLD (ρ = 0.0154, p = 0.898) or cholestatic liver disease (ρ = 0.103, p = 0.750). However, in patients with viral hepatitis, there was a weak negative correlation between CAP and HVPG (ρ = -0.355, p≤0.001). Interestingly, a significant negative correlation was also evident in subgroup analysis according to liver stiffness quartiles: In patients with <12.8kPa, CAP correlated negatively with HVPG (ρ = -0.512, p≤0.001) as well as in patients with 12.8–25.7kPa (ρ = -0.293, p = 0.048). In contrast, no correlation was observed in patients with higher stiffness values (25.8–49.5kPa: ρ = -0.233, p = 0.062; ≥49.6kPa: ρ = 0.028, p = 0.831). This finding was further studied by stratifying patients according to fibrosis stages. While no correlation was observed in patients with F0/1 fibrosis (ρ = 0.113, p = 0.712), a strong negative correlation was found in patients with F2 fibrosis (ρ = -0.586, p = 0.003) and F3 (ρ = -0.543, p = 0.013). Again, the correlation was considerably weaker in patients with LSM indicative for cirrhosis (F4, ρ = -0.139, p = 0.047).

### Severity of portal hypertension according to hepatic steatosis grade (Fig 2)

**Fig 2:**
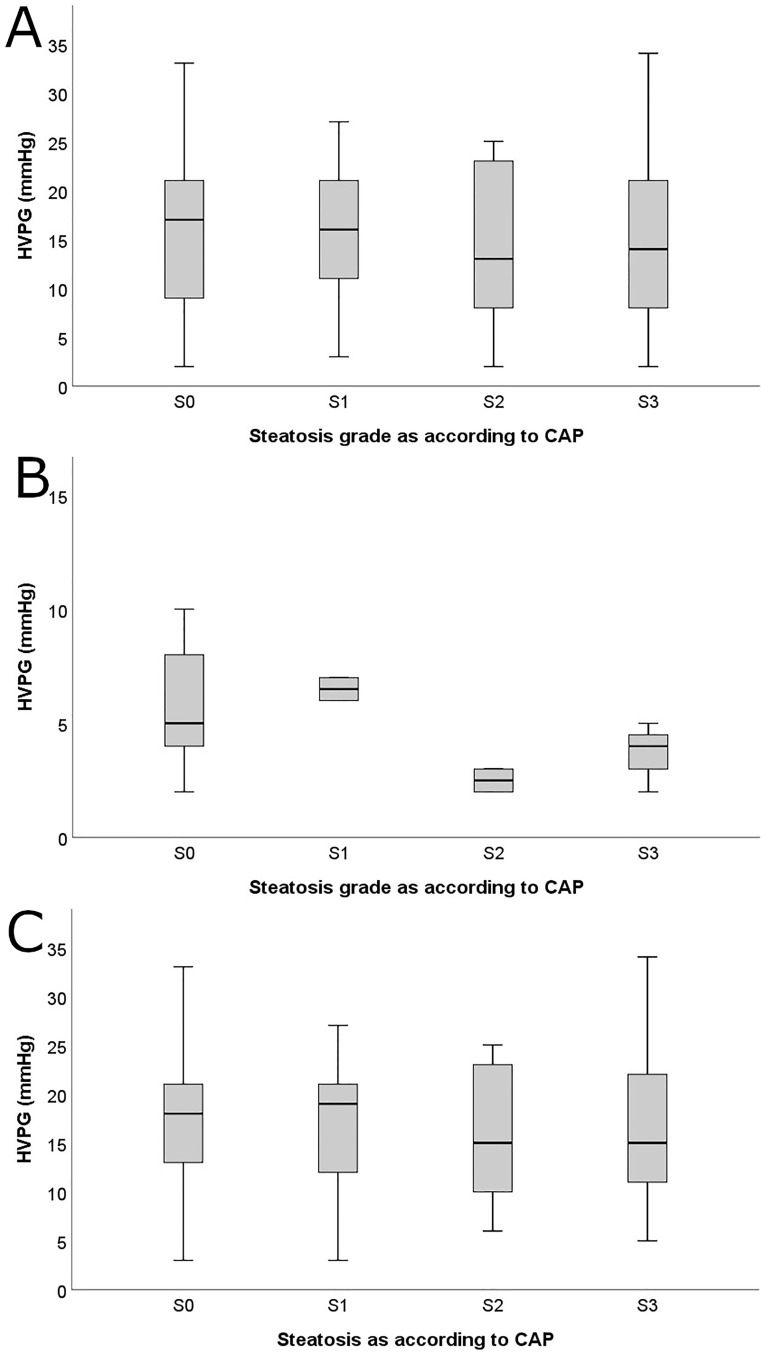
HVPG values according to hepatic steatosis grades (as assessed by CAP) in the (A) overall cohort, (B) patients with F0–2 fibrosis, and (C) F3/4 fibrosis.

HVPG values were comparable between patients with no, mild, moderate and severe steatosis in the overall cohort (HVPG in S0: 15.5±7.5mmHg vs. S1: 15.9±7.2mmHg vs. S2: 14.5±7.5mmHg vs. S3: 14.4±7.9mmHg, p = 0.753). Similarly, when stratifying the cohort into patients with F0-F2 (S0: 6.6±5.4mmHg vs. S1: 6.5±0.7mmHg vs. S2: 2.5±0.7mmHg vs. S3: 3.8±1.0mmHg, p = 0.351) and patients with F3-F4 (S0: 17.1±6.6mmHg vs. S1: 16.8±6.9mmHg vs. S2: 15.8±6.8mmHg vs. S3: 16.2±7.2mmHg, p = 0.779), the HVPG values did not differ across steatosis grades.

### Level of CAP within different stages of liver stiffness and HVPG

CAP values were comparable across liver stiffness quartiles (<12.8kPa: 226±58dB/m vs. 12.8–25.7kPa: 246±65dB/m vs. 25.8–49.5kPa: 230±51dB/m vs. ≥49.6kPa: 247±60dB/m, p = 0.121) as well as between patients with CSPH (235±58dB/m) and those without (242±53dB/m, p = 0.395, S2 Fig).

### Factors independently associated with the presence of CSPH (Table 3)

**Table 3:** Binary logistic regression analysis of factors associated with the presence of CSPH.

	Univariate analysis			Multivariate analysis		
Patient characteristics	OR	95%CI	p-value	OR	95%CI	p-value
**Age, per year**	1.04	1.01–1.06	**0.003**	1.02	0.98–1.07	0.371
**Presence of diabetes mellitus**	3.83	1.31–11.20	**0.014**	3.35	0.54–20.75	0.194
**TE, per kPa**	1.25	1.17–1.34	**<0.001**	1.26	1.17–1.36	**<0.001**
**MELD, per point**	1.40	1.22–1.59	**<0.001**	1.08	0.94–1.24	0.271
**CAP, per 10 dB/m**	0.98	0.93–1.03	0.393	0.92	0.85–1.00	**0.045**

*Abbreviations*: CSPH clinically significant portal hypertension; OR odds ratio; 95%CI 95% confidence interval; TE transient elastography; MELD model for end-stage liver disease; CAP controlled attenuation parameter.

In univariate analysis, older age (OR per year: 1.04, 95%CI: 1.01–1.06, p = 0.003), the presence of diabetes (OR 3.83, 95%CI: 1.31–11.20, p = 0.014), higher liver stiffness (OR per kPa: 1.25, 95%CI: 1.17–1.34, p≤0.001), and higher MELD (OR per point: 1.40, 95%CI: 1.22–1.59, p≤0.001) were associated with the presence of CSPH. While CAP (OR per 10 dB/m: 0.98, 95%CI: 0.93–1.03, p = 0.393) was not associated with CSPH in univariate analysis, after correcting for the above-mentioned factors, a higher CAP (OR per 10dB/m: 0.92, 95%CI: 0.85–1.00, p = 0.045) was an independent protective factor for the presence of CSPH in multivariate analysis. Moreover, only higher TE (OR per kPa: 1.26, 95%CI: 1.17–1.36, p≤0.001) was an additional independent risk factor for CSPH.

### Comparison of HVPG with histological steatosis grade

In 78 patients (29.9%), histological data was available. In these patients, CAP significantly correlated with histological steatosis (Pearson’s r = 0.355, p = 0.001). However, there was neither a correlation of the percentage of histological steatosis with HVPG (Spearman’s ρ = -0.042, p = 0.714) nor of the histological steatosis grade (Spearman’s ρ = -0.007, p = 0.957). Additionally, subgroup analyses among different etiologies, histological fibrosis stages and fibrosis stages determined by TE did not reveal a significant correlation except for a trend towards a negative correlation of HVPG with hepatic steatosis in patients with cirrhosis on liver histology (Spearman’s ρ = -0.342, p = 0.054; S1 Table).

## Discussion

Hepatic fibrosis has been widely acknowledged as the main ‘mechanical’ contributor to PHT, due to its profound impact on the structural component of intrahepatic resistance. However, some animal studies have recently been investigating the influence of hepatic steatosis on the development and progression of PHT.[12–16] Portal pressure was found to be increased in the absence of liver fibrosis in three rat-models with diet-induced NAFLD. Furthermore, these results were confirmed in a small study on humans including 50 patients with NAFLD, of which 14 patients had elevated HVPG (≥6mmHg).[12, 14–16] However, we could not confirm these findings in our study using CAP as a validated non-invasive marker for hepatic steatosis. In our cohort, we neither observed a significant correlation between CAP and HVPG in the overall study population, nor in the subgroups of patients with or without CSPH. This finding indicates that hepatic steatosis is no major factor contributing to portal venous pressure in patients with chronic liver disease. In multivariate analysis, after correcting for other factors potentially associated with CSPH, such as age, the presence of diabetes[41], liver stiffness, and MELD score, higher CAP values were even protective for the presence of CSPH. Of note, the majority of our patients had advanced chronic liver disease (ACLD) as indicated by a median liver stiffness of 28.0kPa. Importantly, previous studies have demonstrated the disappearance of hepatic steatosis with liver fibrosis progression in NAFLD patients (i.e. ‘burnt-out NASH’)[42], a phenomenon which might also occur in other etiologies of ACLD and therefore may have potentially attenuated the association between CAP and HVPG in our study cohort of ACLD patients. Of note, hepatic steatosis on histology was neither correlated with HVPG in the overall cohort nor in subgroup analyses, despite a trend towards a negative correlation in patients with cirrhosis. Although this finding would suggest a ‘protective’ effect of hepatic steatosis in these patients, the low sample size (n = 36) limits this finding. Noteworthy, the above-mentioned animal studies need to be interpreted with caution. Since these animals developed very pronounced hepatic steatosis in a short period of time, it is unclear whether these findings can be extrapolated to humans, in whom lifestyle-induced hepatic steatosis persists over a long period of time and is substantially less pronounced. Thus, these experimental studies might considerably overestimate the relevance of hepatic steatosis in the human setting. Moreover, animals, if at all, only showed mild hepatic inflammation/liver fibrosis.[12, 14] In our clinical setting, other factors contributing to portal hypertension, such as increases in intrahepatic resistance due to hepatic inflammation/fibrosis, as well as splanchnic vasodilatation and the hyperdynamic circulation in patients with CSPH might have had a much stronger impact on portal pressure than hepatic steatosis itself.[43] So far, only one human study evaluated the effect of simple hepatic steatosis on HVPG in humans and found a significant positive correlation between hepatic steatosis and HVPG, however, this study only included 14 patients with elevated HVPG with a mean pressure of 8.8 ± 2.6mmHg and only one patient had liver cirrhosis.[16] This is in contrast to our study population with most patients having progressed to cirrhosis and three quarters of patients having CSPH. Insulin resistance (IR) is an important driver for the development of portal hypertension and hepatic steatosis.[44] Thus, in patients where IR is the key driver of steatosis, IR-induced endothelial dysfunction might aggravate sinusoidal vasoconstriction and therefore contribute to the development of early portal hypertension. However, this factor does not seem to have a strong impact on HVPG in patients with ACLD and established PHT. Interestingly, we observed a significant negative correlation between HVPG and CAP in various subgroups, especially in patients with liver fibrosis stages F2/3, as assessed by TE. One explanation could be an ‘artificial’ increase of TE-based liver stiffness in these patients by pronounced hepatic steatosis, resulting in an overestimation of liver fibrosis. Recently, there has been a controversy regarding the impact of CAP on the association between LSM and fibrosis[45, 46]. Petta et al. (2015)[45] observed increased LSM in patients with F0–2 in patients with severe steatosis. Furthermore, the same group reported a higher false positive rate for the diagnosis of liver fibrosis stage F3/4 and also decreasing diagnostic accuracy for discriminating F0–2 from F3–4 among patients with advanced steatosis according to CAP values.[46] As a result, the ‘true’ liver fibrosis stage as assessed by histology would be lower in our study, which could explain the indirect correlation between CAP and HVPG values where HVPG was lower than expected from LSM values. This study has several limitations that need to be considered when interpreting its results: First, nearly half of the patients undergoing HVPG and CAP measurements needed to be excluded due to potential confounding factors such as antiviral therapy or non-selective beta-blocker therapy, which left a limited number of patients for the final analyses. Secondly, the majority of our patients had other etiologies than (N)AFLD and suffered from ACLD representing a selection bias towards high proportions of patients with cirrhosis and CSPH in whom hepatic steatosis may be of limited relevance for PHT. Moreover, the proportions of patients with moderate or severe steatosis was limited. In the early study period only the M-probe was available to obtain CAP, which leaves the possibility of elevated LSM and CAP values in obese patients. It has to be considered that CAP might have limited diagnostic value for assessing hepatic steatosis in patients with ACLD.[47, 48] Finally, hepatic steatosis might imply a certain degree of presinusoidal PHT through hepatocyte ballooning and sinusoidal distortion, which may not be fully captured by HVPG measurement. This limitation could only be overcome by direct measurement of portal pressure (gradient), which is rarely performed unless the patient is undergoing TIPS. However, as all ACLD patients undergoing TIPS have CSPH and even more advanced disease than our cohort, this would lead to an even stronger selection towards patients in whom the effect of hepatic steatosis on PHT is expected to be less pronounced. In conclusion, hepatic steatosis—as assessed by CAP—was not associated with an increase in portal pressure in our cohort comprising a high proportion of patients with ACLD. However, hepatic steatosis might lead to overestimation of liver fibrosis by ‘artificially’ increasing TE-based LSM.

## Supporting information

S1 Fig Patient flow-chart showing the number of in- and excluded patients. (DOCX)

S2 Fig CAP values in different liver stiffness quartiles. (DOCX)

S1 Table Correlation between CAP and histological steatosis grade in (A) different etiologies of liver disease, (B) subgroups of different fibrosis stages (as according to transient elastography) and (C) subgroups of different fibrosis stages according to liver histology. (DOCX)

## Abbreviations

ACLD: advanced chronic liver disease
BMI: body mass index
CAP: controlled attenuation parameter
CI: confidence interval
CSPH: clinically significant portal hypertension
HCC: hepatocellular carcinoma
HVPG: hepatic venous pressure gradient
IQR: interquartile range
IR: insulin resistance
LSM: liver stiffness measurement
MCDD: methionine-choline-deficient diet
MELD: model of end stage liver disease
NAFLD: non-alcoholic fatty liver disease
OR: odds ratio
PHT: portal hypertension
SD: standard deviation
TE: transient elastography
TIPS: transjugular intrahepatic portosystemic shunt
