# Supplementary material for: The impact of hepatic steatosis on portal hypertension
Source: PLoS One. 2019 Nov 6;14(11):e0224506. doi: 10.1371/journal.pone.0224506 (PMC6834246; doi:10.1371/journal.pone.0224506)
Supplement: S1 Fig — (DOCX) [file pone.0224506.s001.docx]

**S1 Fig.** Patient flow-chart showing the number of in- and excluded patients.
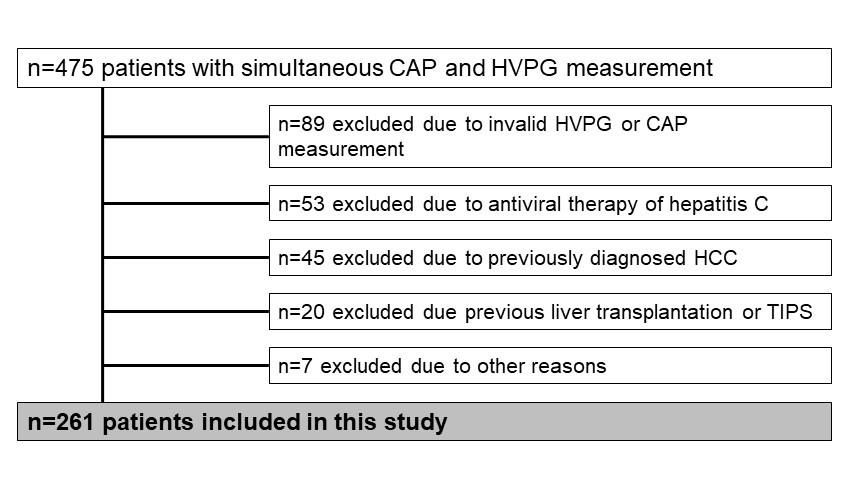


*Abbreviations: CAP controlled attenuation parameter; HVPG hepatic venous pressure gradient; HCC hepatocellular carcinoma; TIPS transjugular intrahepatic portosystemic shunt*
