# Supplementary figures and images for: The impact of hepatic steatosis on portal hypertension
Source: PLoS One. 2019 Nov 6;14(11):e0224506. doi: 10.1371/journal.pone.0224506 (PMC6834246; doi:10.1371/journal.pone.0224506)

**S2 Fig.** CAP values in different liver stiffness quartiles.


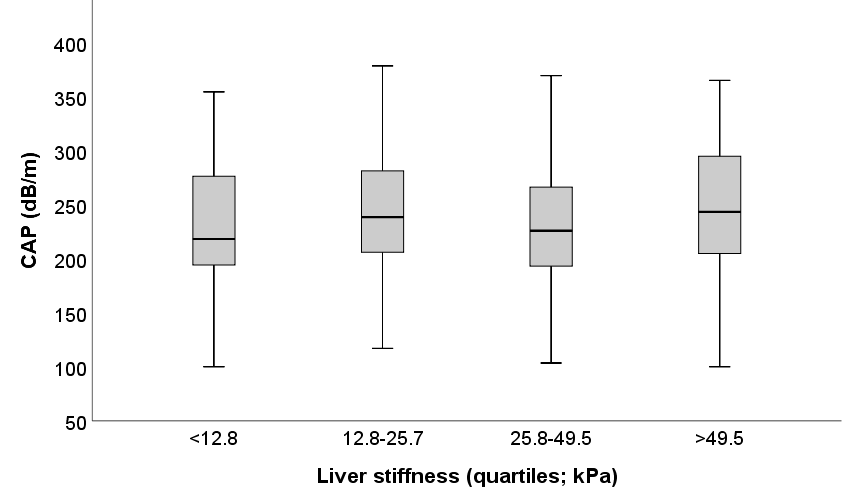

Supplement: S2 Fig — (DOCX) [file pone.0224506.s002.docx]
