# Supplementary material for: The impact of hepatic steatosis on portal hypertension
Source: PLoS One. 2019 Nov 6;14(11):e0224506. doi: 10.1371/journal.pone.0224506 (PMC6834246; doi:10.1371/journal.pone.0224506)
Supplement: S1 Table — (DOCX) [file pone.0224506.s003.docx]

**S1 Table.** Correlation between CAP and histological steatosis grade in **(A)** different etiologies of liver disease, **(B)** subgroups of different fibrosis stages (as according to transient elastography) and **(C)** subgroups of different fibrosis stages according to liver histology.

**A**

| **HVPG – hepatic steatosis (%)** | **Correlation coeff.**  **(Spearman)** | ***p-*value** |
| --- | --- | --- |
| **(N)AFLD (n=13)** | -0.008 | 0.978 |
| **Cholestatic liver disease (n=3)** | - | - |
| **Viral liver disease (n=26)** | -0.304 | 0.131 |
| **Other etiologies of liver disease (n=15)** | 0.242 | 0.385 |

**B**

| **HVPG – hepatic steatosis (%)** | **Correlation coeff. (Spearman)** | ***p-*value** |
| --- | --- | --- |
| **F0/1 (n=17)** | -0.242 | 0.349 |
| **F2 (n=15)** | -0.017 | 0.951 |
| **F3 (n=20)** | 0.193 | 0.648 |
| **F4 (n=36)** | -0.342 | 0.054 |

**C**

| **HVPG – hepatic steatosis (%)** | **Correlation coeff. (Spearman)** | ***p-*value** |
| --- | --- | --- |
| **F0/1 (n=2)** | - | - |
| **F2 (n=9)** | -0.033 | 0.934 |
| **F3 (n=10)** | -0.069 | 0.860 |
| **F4 (n=58)** | -0.004 | 0.976 |
| **F2/F3 (n=19)** | -0.142 | 0.562 |
